# Supplementary material for: Causal Relationship between Aging and Anorexia Nervosa: A White-Matter-Microstructure-Mediated Mendelian Randomization Analysis
Source: Biomedicines. 2024 Aug 16;12(8):1874. doi: 10.3390/biomedicines12081874 (PMC11351342; doi:10.3390/biomedicines12081874)
Supplement: Supplementary file 1 [file biomedicines-12-01874-s001.zip › Supplementary Figure/Figure S4/Singe SNP plot of MR analysis for TL to ubm-b-1794.pdf]

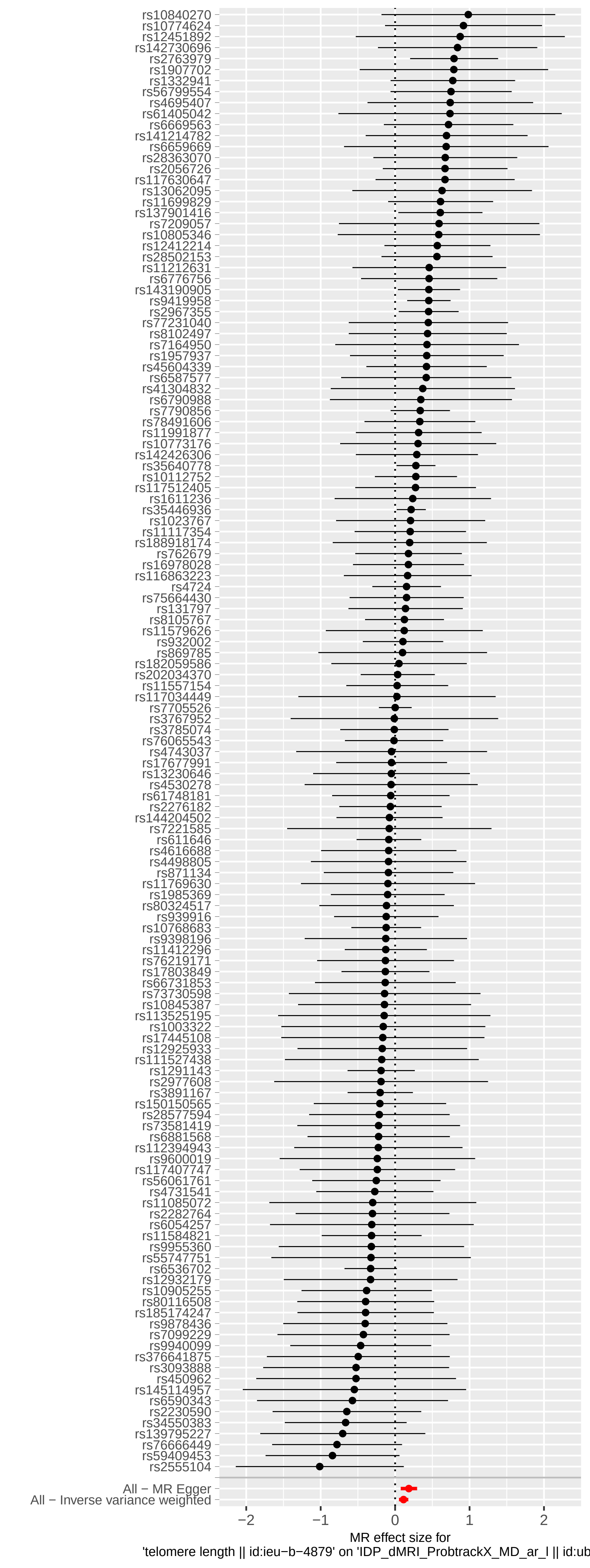

rs10840270
rs10774624
rs12451892
rs142730696
rs2763979
rs1907702
rs1332941
rs56799554
rs4695407
rs61405042
rs6669563
rs141214782
rs6659669
rs28363070
rs2056726
rs117630647
rs13062095
rs11699829
rs137901416
rs7209057
rs10805346
rs12412214
rs28502153
rs11212631
rs6776756
rs143190905
rs9419958
rs2967355
rs77231040
rs8102497
rs7164950
rs1957937
rs45604339
rs6587577
rs41304832
rs6790988
rs7790856
rs78491606
rs11991877
rs10773176
rs142426306
rs35640778
rs10112752
rs117512405
rs1611236
rs35446936
rs1023767
rs11117354
rs188918174
rs762679
rs16978028
rs116863223
rs4724
rs75664430
rs131797
rs8105767
rs11579626
rs932002
rs869785
rs182059586
rs202034370
rs11557154
rs117034449
rs7705526
rs3767952
rs3785074
rs76065543
rs4743037
rs17677991
rs13230646
rs4530278
rs61748181
rs2276182
rs144204502
rs7221585
rs611646
rs4616688
rs4498805
rs871134
rs11769630
rs1985369
rs80324517
rs939916
rs10768683
rs9398196
rs11412296
rs76219171
rs17803849
rs66731853
rs73730598
rs10845387
rs113525195
rs1003322
rs17445108
rs12925933
rs111527438
rs1291143
rs2977608
rs3891167
rs150150565
rs28577594
rs73581419
rs6881568
rs112394943
rs9600019
rs117407747
rs56061761
rs4731541
rs11085072
rs2282764
rs6054257
rs11584821
rs9955360
rs55747751
rs6536702
rs12932179
rs10905255
rs80116508
rs185174247
rs9878436
rs7099229
rs9940099
rs376641875
rs3093888
rs450962
rs145114957
rs6590343
rs2230590
rs34550383
rs139795227
rs76666449
rs59409453
rs2555104
All − MR Egger
All − Inverse variance weighted
−2
−1
0
1
2
MR effect size for
'telomere length || id:ieu−b−4879' on 'IDP_dMRI_ProbtrackX_MD_ar_l || id:ub
